# New insights into prediction of weak π–π complex association through proton-nuclear magnetic resonance analysis

**DOI:** 10.1186/s13065-020-00718-x

**Published:** 2020-10-30

**Authors:** Chenyu Lin, Joseph Skufca, Richard E. Partch

**Affiliations:** 1 Chemistry & Biomolecular Department, Clarkson University, Potsdam, NY 13699 USA; 2 Mathematics Department, Clarkson University, 8 Clarkson Ave., Potsdam, NY 13699 USA

**Keywords:** pi–pi interaction, Supramolecular complexes, Additional unspecific shielding effects, Ring current effects, Complex geometries and stabilities

## Abstract

For analysis of weak *π*–*π* complexes proton-nuclear magnetic resonance (proton-NMR) simultaneously provides information of stacking configurations and association constants $\left( K \right)$ However, an apparent issue for this approach is inconsistent/impossible constant estimation which often leads to unreasonable interpretation for *π*–*π* complexation. Whether or not this proton-dependent constant variation could be attributed to simple experimental uncertainties or to more sophisticated additional unspecific shielding effects (AUS effects) was addressed by means of hypothesis tests using a robust bootstrap technique in this report. Our analysis shows the significance of AUS effects on such variation in constant estimation. A following study using numeric simulation further reveals the variation patterns induced by AUS effects and concludes that the largest $K$ among the obtained $K$ estimates of a complex is considered as the best estimate of $K$ due to minimum deviation from the true value of *K* and the multiple $K$ estimates of a *π*–*π* complex could provide preferable inferences for complex geometries.

## Introduction

Self-assembled $\pi$ electron acceptor–donor molecular complexes (*π*–*π* complexes) have been intensively studied in broad fields including conformational structures of biomolecules like DNA, RNA and proteins [1–7], and design and quantitative analyses [8–12] for drug overdose remediation [13–15]. *π*–*π* complexes usually involve complexation between a $\pi$ electron donor and a $\pi$ electron acceptor. Understanding complex stabilities and geometries are critically important when *π*–*π* complexes are designed and interpreted.

When investigating weak *π*–*π* complexes, proton-NMR is the most important approach not only due to its simple and rapid analysis process but due to simultaneously estimation of both stabilities and geometries for an *π*–*π* complex in solvents. However, an often encountered issue for this proton-NMR-based approach is that the estimates of association constants ($K$) vary depending on which acceptor protons of the tested *π*–*π* complex are observed [13, 16]. For 1:1 acceptor to donor *π*–*π* complexes, upfield shifts of observed protons of an acceptor are historically assumed to result only from ring current effects of the stacking donor [17]. Therefore, the estimates of $K$ from the NMR data obtained at the protons that sense the ring current effects are conventionally treated identical and the estimates of complex shifts (${\Delta }_{C}$, the difference between chemical shifts of observed acceptor protons in complexed forms and in uncomplexed forms) depend on the geometric positions of observed protons [17]. In other words, the difference in the $K$ values of a *π*–*π* complex is simply ascribed to experimental errors and an average $K$ value is often selected as a representative association constant for *π*–*π* complexes [18]. It means that $K$ values are proton independent and the experimentally obtained various upfield shifts (${\Delta }$, the difference between the chemical shifts of acceptor protons in the absence and in the presence of donors) primarily result from the difference in ${\Delta }_{C}$ induced by the offset geometry of a complex [17, 19–21].

Despite that the statistical analysis adopted in those historical data treatments is not clarified, we assumed that t-test is most likely used for the treatment of observed different $K$ estimates. In fact, we did hypothesis tests using t-test for our experimental data of *π*–*π* complexes and obtained the results in accord with those literature results. However, using t-test relies on the assumption of sampling distribution normality of estimates, [22] which might not always hold when both constant estimates are obtained from more complicated mathematic models [23]. Therefore, in this report, we use a robust statistical bootstrap analysis which is expected to provide more reliable judgments on the hypothesis test results. Our statistical analysis suggests that such difference in constant estimates may not be simply ascribed to experimental errors but to additional factors that cause such apparent different values. We adopt a theory, additional unspecific shielding effects (AUS effects), put forward by Stamm et al. [24–28] to interpret such difference and use numerical simulation to investigate the impact of AUS effects on $K$ and ${\Delta }_{C}$ estimates. The results are presented in “Results” section. The numeric simulation provides useful information for treatment of inconsistent $K$ estimates and a new approach for geometric inferences without the need of changing experimental conditions. The results and the complex geometric inferences are presented in “Discussion” section.

## Experimental

### Material

**Fig. 1.**
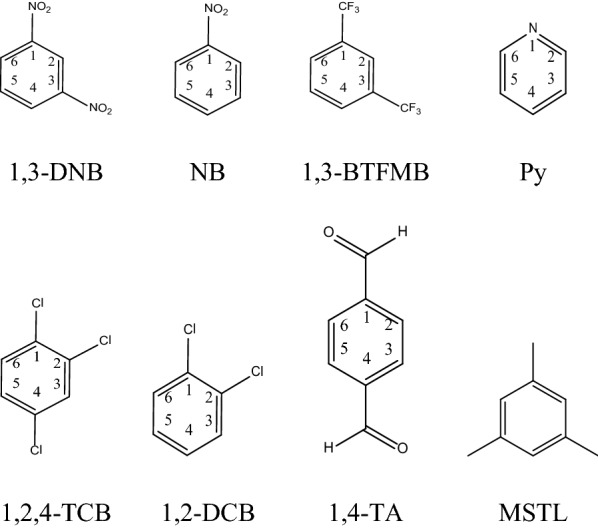
The structures of seven aromatic molecules (acceptors) used to interact with MSTL

The following chemicals were used for *π*–*π* complexation. $\pi$ electron acceptors were 1,3-dinitrobenzene (1,3-DNB, 97%, Sigma-Aldrich); nitrobenzene (NB, 99%, Acros); 1,3-bis(trifluoromethyl)benzene (1,3-BTFMB, 98%, Sigma-Aldrich); 1,2,4-trichlorobenzene (1,2,4-TCB, 99%, Sigma-Aldrich); 1,2-dichlorobenzene (1,2-DCB, 98.6%, Sigma-Aldrich); terephthalaldehyde (TA, 99%, Sigma-Aldrich); pyridine (Py, 99% Sigma-Aldrich) $\pi$ electron donor was mesitylene (MSTL, 97%, JT Baker). Structures for all acceptors and donor are shown in Fig. 1. The solvent was cyclohexane (99%, Acrose).

### Proton nuclear magnetic resonance (proton-NMR)

Bruker 400 MHz NMR was used to obtain ^1^H spectra for all species in the complexation reactions. For the use of cyclohexane solvent, manual shimming was carried out without lock. Each sample was scanned for 30 times with 30° pulse angle and 3 s for relaxation delay. Chemical shifts of acceptor protons were reported in parts per million (ppm) with respect to that of internal standard, tetramethylsilane (TMS). The readings of chemical shifts were analyzed using MestRC program.

### Experimental procedure

The experiments regarding *π*–*π* complexation were performed by the following procedure: each quantity was carefully measured using an electric balance with four decimal digit readings. Following the suggestions by Kuntz et al. [29] we used molarity as the concentration scale. The stock solutions of acceptors were first prepared in cyclohexane with the concentration of 0.02 M. For noncomplexed acceptor solutions, an aliquot of a stock solution was added in 2 ml volumetric flask, followed by the addition of 0.1 ml tetramethylsilane (TMS) and by filling the flask with cyclohexane to the mark. The acceptor concentrations were made just large enough (usually around 0.001–0.01 M) to be observed by NMR spectroscopy. For complexation reactions, a series of solutions for NMR detection were prepared with small acceptor concentration (same as the noncomplexed acceptor solutions) and the various excess MSTL concentrations (0.1–0.9 M) in cyclohexane. To make a pi–pi complexation solution, a small aliquot of the acceptor, the desired amount of the donor and 0.1 ml TMS were then added into a 2 ml volume flask followed by filling the flask with cyclohexane to the 2 ml mark. After mixing, the solution was transferred to a 5 ml glass vial and capped tightly for 2 h. Afterward, the solution was transferred to an NMR tube for proton NMR (proton-NMR) measurements.

### Individual curve fitting for ${\mathbf{K}}$ and ${{\varvec{\Delta}}}_{{\mathbf{C}}}$ estimates

Curve fitting is suggested to provide more reliable $K$ estimates than other linear regressions [30, 31]. The curve fitting Eq. 1 is performed in Originpro to individually analyze the upfield shift data of each acceptor proton to estimate $K$ and ${\Delta }_{C}$. The equation for the individual curve fitting is presented as.

1 $$ \Delta = \frac{{\left( {Ka_{0} + Kd_{0} + 1} \right) - \sqrt {\left( {Ka_{0} + Kd_{0} + 1} \right)^{2} - 4K^{2} a_{0} d_{0} } }}{{2a_{0} K}}\Delta_{{\text{C}}} , $$

where $a_{0}$ and $d_{0}$ denote initial concentrations of acceptors and donors, respectively. $\Delta = \delta_{A} - \delta \;{\text{and}}\;\Delta_{{\text{C}}} = \delta_{A} - \delta_{{\text{C}}}$ where $\delta$ is the observed acceptor chemical shifts in the presence of donors; $\delta_{A}$ and $\delta_{C}$ are the chemical shifts of the acceptor protons in the noncomplexed form and in the complexed form, respectively.

### Hypothesis tests

Our two-tailed hypothesis test followed the procedure suggested by Mann [22]. We analyzed differences in both $K$. and ${\Delta }_{C}$ estimates using hypothesis testing. For ease of notation, we used $H_{i} = H_{j}$ to denote null hypothesis $H_{NULL} :\left( {K_{i} = K_{j} } \right)$ or $H_{NULL} :({\Delta }_{Ci} = {\Delta }_{Cj}$) where $K_{i}$ and ${\Delta }_{Ci} { }$ are the values computed for the ith proton. We use $H_{i} \ne H_{j}$ to denote the associated pairs of alternative hypothesis, $H_{ALT} :\left( {K_{i} \ne K_{j} } \right)$ d $H_{ALT} :\left( {{\Delta }_{i} \ne {\Delta }_{j} } \right)$. Subscripts $i$ and $j$ denote the proton positions as indicated in Fig. 1. For chemically equivalent protons the lowest numbered protons are used in all tables.

The hypothesis tests use a bootstrap technique that approximates the sampling distributions of $K$ and ${\Delta }_{C}$ estimates [32]. We adopted fixed x resampling following the procedure suggested by Fox [32]. The resampling processes for each complex constants were programmed in Maple 17 with the resampling number, 10,000 times. The resulting bootstrap distributions were tested by the Anderson–Darling method for the normality tests [23]. When the normality of the bootstrap distributions is accepted, the following hypothesis tests are performed using normal distribution [22] and the significant level $\alpha = 0.05/n$ (Bonferroni’s suggestion) [23]. When the normality is rejected, the comparisons of obtained bootstrap distributions are performed using the procedure of nonparametric Mann Whitney test (M–W test) [23] and $\alpha = 0.05/n$ are used when there are two or more bootstrap distributions to compare.

## Results

### Results of hypothesis tests for ${\varvec{K}}$ and ${{\varvec{\Delta}}}_{{\varvec{C}}}$ estimates

**Fig. 2:**
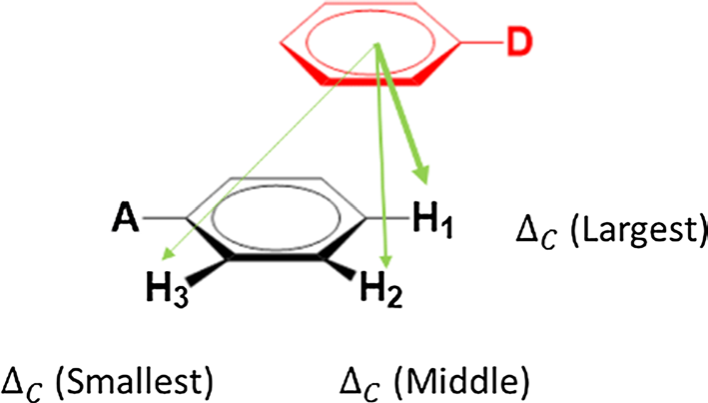
The relationship between ${{\varvec{\Delta}}}_{{\varvec{C}}}$ and the geometric positions of acceptor protons in a ***π***–***π*** complex. The green arrows refer to the shielding caused by the ring current effect of the complexed donor. The protons closer to the donor ring center sense greater ring current effect (thicker green arrows) and exhibit larger ${{\varvec{\Delta}}}_{{\varvec{C}}}$

Weak *π*–*π* complexes often show offset geometries, Fig. 2 [33, 34]. When proton-NMR is used for analysis of such *π*–*π* complexes, upfield shifts of observed protons are often treated as a characteristic of complexation. Indeed, all protons in our experiments exhibited upfield shifts suggesting the formation of *π*–*π* complexation. The estimates of $K$ and ${\Delta }_{C}$ for each kind of protons were positive with $R^{2} \ge 0.999$ and all values with their standard errors (SE) are presented in Table 1.

**Table 1:** Estimates of ${\varvec{K}}$ (${\text{M}}^{ - 1}$) and ${{\varvec{\Delta}}}_{{\varvec{C}}}$ (ppm) and their standard errors (SE) for all protons of acceptors

Acceptors	Protons	$K$	SE	${\Delta }_{C}$	SE
1,3-DNB	H_2_	0.769	0.024	0.843	0.019
	H_4_	0.707	0.017	0.928	0.017
	H_5_	0.760	0.021	1.05	0.022
NB	H_2_	0.293	0.019	0.595	0.034
	H_3_	0.277	0.019	0.774	0.045
	H_4_	0.259	0.020	0.757	0.052
1,2,4-TCB	H_3_	0.175	0.011	0.808	0.047
	H_5_	0.204	0.018	0.969	0.077
	H_6_	0.221	0.012	0.939	0.045
1,2-DCB	H_3_	0.186	0.023	0.634	0.067
	H_4_	0.179	0.017	0.756	0.069
1,3-BTFMB	H_2_	0.283	0.045	0.233	0.032
	H_4_	0.320	0.013	0.671	0.023
	H_5_	0.324	0.012	0.892	0.027
TA	H_Rign_	0.390	0.019	0.786	0.034
	H_Ald_	0.379	0.020	0.843	0.038
Py	H_2_	0.070	0.023	0.732	0.227
	H_3_	0.063	0.005	1.384	0.107
	H_4_	0.082	0.009	1.191	0.127

The variation in the estimates of $K$ and ${\Delta }_{C}$ given a *π*–*π* complex is statistically analyzed using the bootstrap technique for hypothesis tests. The results are presented in Table 2. In Table 2 we can observe that null hypothesis test results for $K$ and ${\Delta }_{C}$ are all rejected, except for $K$ estimates at HA and HAld of TA and for ${\Delta }_{C}$ estimates at H5 and H6 of 1,2,4-TCB. The evidence suggests a significant difference in those constant estimates obtained from different protons of a *π*–*π* complex. For those which show the results “accept,” we do not have strong evidence to distinguish these estimated values from one another. The absence of the significant difference among these tested quantities does not necessarily mean that they are not different, but that further analysis may be required.

**Table 2:** Hypothesis tests for complex constants

Acceptors	Null	$K$		${\Delta }_{C}$	
		P-value	Result	P-value	Result
1,3-DNB	H_2_ = H_4_	0	Reject	0	Reject
	H_2_ = H_5_	0	Reject	0	Reject
	H_4_ = H_5_	0	Reject	0	Reject
1,3-BTFMB	H_2_ = H_4_	0	Reject	0	Reject
	H_2_ = H_5_	0	Reject	0	Reject
	H_4_ = H_5_	0	Reject	0	Reject
NB	H_2_ = H_3_	0	Reject	0	Reject
	H_2_ = H_4_	0	Reject	0	Reject
	H_3_ = H_4_	0	Reject	0	Reject
1,2,4-TCB	H_3_ = H_5_	0	Reject	0	Reject
	H_3_ = H_6_	0	Reject	0	Reject
	H_5_ = H_6_	0	Reject	0.377	Accept
1,2-DCB	H_3_ = H_4_	0	Reject	0	Reject
TA	H_Ring_ = H_Ald_	0.902	Accept	0	Reject
Py	H_2_ = H_3_^(a)^	0	Reject	0	Reject
	H_2_ = H_4_	0	Reject	0	Reject
	H_3_ = H_4_	0	Reject	0	Reject

The null hypothesis: ${\varvec{H}}_{{\varvec{i}}} = {\varvec{H}}_{{\varvec{j}}}$ and alternative hypothesis: ${\varvec{H}}_{{\varvec{i}}} \ne {\varvec{H}}_{{\varvec{j}}}$. α is set as 0.05/n. where n is the number of the tested groups. The P-values that are too small to present are denoted as “0” in the table

^(a)^The bootstrap distributions for ortho protons (H2 and H6) of Py exhibit the negative percentile range, − 0.058 and − 0.017 ${\text{M}}^{ - 1}$ for $K$ and the range, − 2.83 and − 0.81 ppm for ${\Delta }_{C}$

The statistical analysis suggests that the difference in these constant estimates may not be simply attributed to experimental uncertainties, which partially disagrees with the assumption of the model shown in Fig. 2 [17, 34]. In most cases, this model simply attributes the difference in ${\Delta }$. values to the difference in ${\Delta }_{C}$ values because of offset geometries but $K$ estimates at different protons are considered identical within experimental errors. In fact, in some extreme offset *π*–*π* complex geometries, this model does allow different $K$ estimates at the protons which are far away from the stacking donor but their corresponding ${\Delta }_{C}$ should be small. However, ${\Delta }_{C}$ estimates at such protons are always largest among the estimates, which contradictorily implies the shortest distance between the protons and the stacking donors. Therefore, this model is not sufficient to interpret the significant difference in our $K$ estimates.

In order to interpret our statistical results, we adopted the theory of additional unspecific shielding effects (AUS effects) to understand the potential factors responsible for the significant difference in $K$ estimates. AUS effects are put forward by Stamm, et al. [24] to explain unexpected curve distributions of proton-NMR data in the plots which should give linear distributions. Laszlo and Engler also use a similar concept to study the interactions between camphor and aromatic modelcules [35]. AUS effects are important for *π*–*π* interaction systems especially for donors with excessive concentrations as compared with acceptors (100- to 900-fold in this case). Due to only a small portion of donors complexed by acceptors, a relatively large number of free donors are capable of providing non-complexing collisions to the protons of acceptors in complexed form and in uncomplexed form, which result in additional upfield shifts to those induced by complexation. This concept may also correspond to Orgel and Mulliken’s theory that noncomplexing contact of donors to charge transfer complexes causes additional absorption in UV–VIS spectra [36, 37]. To understand the impact of AUS effects in our system, we conducted a numeric simulation presented as the following.

### Numeric simulation for the impact of AUS effects on ${\varvec{K}}$ and ${\varvec{\Delta}}_{{\varvec{C}}}$ estimates

**Fig. 3:**
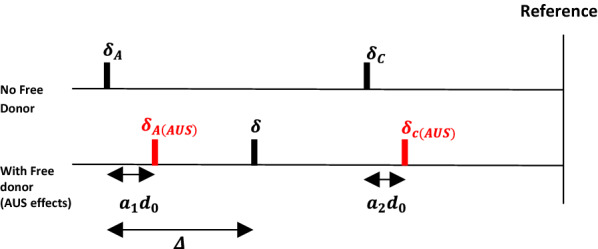
Adapted schematic illustration of Stamm’s AUS effects for NMR chemical shifts [24]. ${\varvec{\delta}}_{{\varvec{A}}}$ and ${\varvec{\delta}}_{{\varvec{C}}}$ are the chemical shifts of non-complexed acceptor protons and acceptor protons in complexes. ${\varvec{\delta}}_{{{\varvec{A}}\left( {{\varvec{AUS}}} \right)}}$ and ${\varvec{\delta}}_{{{\varvec{C}}\left( {{\varvec{AUS}}} \right)}}$ are the chemical shifts affected by AUS effects

Stamm et al. introduce two coefficients, $a_{1}$ and $a_{2}$, for the AUS effects to noncomplexed acceptors and complexed acceptors, respectively, Fig. 3 [24]. The extent of AUS effects on chemical shifts of these protons is defined as $a_{1} d_{0}$ and $a_{2} d_{0}$, respectively. When $d_{0} > 0$ where free donors exist, $\delta_{A}$ and $\delta_{C}$ shift upfield by AUS effects and become $\delta_{{A\left( {AUS} \right)}}$ and $\delta_{{C\left( {AUS} \right)}}$. The $\delta$ is not determined by $\delta_{A}$ and $\delta_{C}$ but by $\delta_{{A\left( {AUS} \right)}}$ and $\delta_{{C\left( {AUS} \right)}}$. Therefore, the experimentally obtained ${\Delta }$ actually results from $\delta_{A} - \delta_{{A\left( {AUS} \right)}}$ rather than $\delta - \delta_{A}$ [26].

Based on the AUS model, we introduce $a_{1}$ and $a_{2}$. into Eq. 1 to obtain Eq. 2 (the equation development is presented in Additional file 1). The new equation is expected to be a better mathematical description than Eq. 1 for *π*–*π* complexation which is under influence of AUS effects. Ideally, both $a_{1}$ and $a_{2}$ need to be evaluated for the estimates of $K$ and ${\Delta }_{C}$. In practice, only $a_{2}$ has been reported by Stamm et al. to be experimentally estimated but it requires wide range and large donor concentrations (around $1\;{\text{M}}$ to $10\;{\text{M}}$) [26]. Using such high donor concentrations could raise concerns including invalidation of the terms, $a_{1} d_{0}$ and $a_{2} d_{0}$, the variation of internal reference signal, [26] and the change in solvent properties, which influence $K$. estimates. Moreover, using such high donor concentrations is not viable in many *π*–*π* interaction systems, especially in those with donors that have low solubility in solvents.

2 $$ \Delta = \frac{{\left( {Ka_{0} + Kd_{0} + 1} \right) - \sqrt {\left( {Ka_{0} + Kd_{0} + 1} \right)^{2} - 4K^{2} a_{0} d_{0} } }}{{2a_{0} K}}\left( {\Delta_{C} - a_{1} d_{0} + a_{2} d_{0} } \right) + a_{1} d_{0} . $$

Instead of the attempt to evaluate AUS effects, we conducted a series of systematic numeric simulations to understand the influence of the presence of AUS effects on $K$. and ${\Delta }_{C}$ estimates under genel experimental conditions. We used Eq. 2 to generate the upfield shift data with set values of AUS effects ($a_{1}$, $a_{2}$), reactant concentrations ($a_{0} = 0.001$ and $0.1 \le d_{0} \le 0.9$), $K$ and ${\Delta }_{C}$ that are close to our rimental conditions and used Eq. 1 to analyze the upfield shift data in order to estimate $K$ and ${\Delta }_{C}$. The comparison between the estimated values and the set values should reveal the patterns of how the presence of AUS effects deviate the constant estimates from the true values in real experiments. In this simulation we discussed two directions: one is the individual AUS effects on constant estimation and the other is their collective influence with ring current effects on estimation for offset *π*–*π* complexation, Fig. 2. For the study of individual AUS effects $a_{1} \ge a_{2}$ both should be less or equal to the average $\overline{a}$ (0.088 for MSTL in the system) [27, 38]. Therefore, 0.09 is set as a maximum value for both *a*_1_ and *a*_2_. Then we set $K, \Delta_{C} = 0.8$, *a*_1_ = 0 and $0 \le a_{2} \le 0.09$ or $a_{2} = 0$ and $0 \le a_{1} \le 0.09$ to study the individual influence on *K* and $\Delta_{C}$ estimates. For the collective effects, AUS effects (various $a_{2}$ and fixed $a_{1}$) and ring current effects (various ${\Delta }_{C}$) are considered together with fixed *K* (0.8). The range of set ${\Delta }_{C}$ values represents different degrees of ring current effects form a stacking donor on observed acceptor protons. The larger ${\Delta }_{C}$ values mean closer distances of the acceptor protons to the ring center of the stacking donor.

**Fig. 4:**
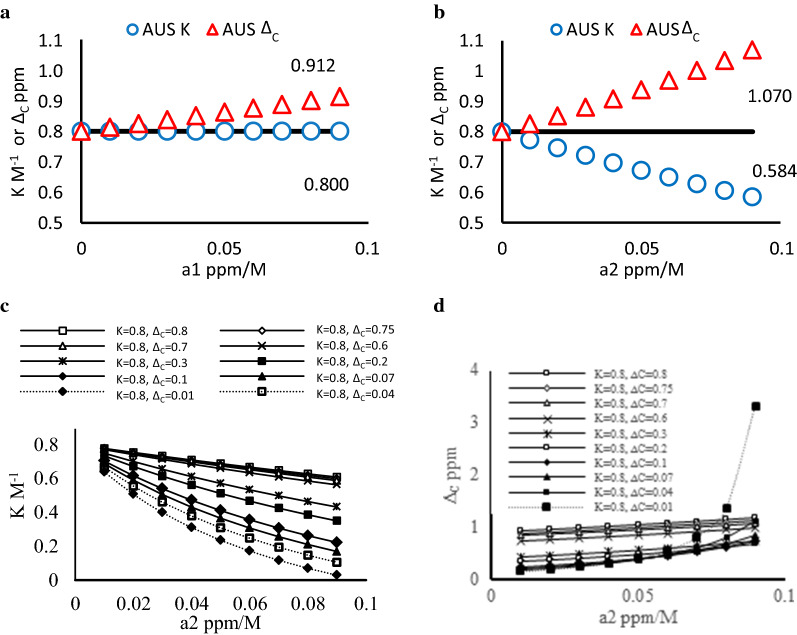
The impact of the AUS effects, ${\varvec{a}}_{1}$ and ${\varvec{a}}_{2}$ , on the estimates of the set ***K*** and ${\Delta }_{C}$. The resulting estimates are AUS ***K*** and AUS ${{\varvec{\Delta}}}_{{\varvec{C}}}$. **a** The study of both AUS ***K*** and AUS ${{\varvec{\Delta}}}_{{\varvec{C}}}$ at various ${\varvec{a}}_{1}$ where ${\varvec{a}}_{2}$ = 0, ***K***, ${{\varvec{\Delta}}}_{{\varvec{C}}}$ = 0.8; **b** the study of both AUS ***K*** and AUS ${\Delta }_{C}$ at various ${\varvec{a}}_{2}$ where ${\varvec{a}}_{1}$ = 0 and ***K***, ${{\varvec{\Delta}}}_{{\varvec{C}}}$ = 0.8; **c** the study of AUS ***K*** at ${\varvec{a}}_{1}$  = 0.09, ${\varvec{a}}_{2}$ = 0.01 – 0.09, ${\varvec{K}} = 0.8$ ${{\varvec{\Delta}}}_{{\varvec{C}}}$ = 0.8–0.01; **d** the study of AUS ${{\varvec{\Delta}}}_{{\varvec{C}}}$ with the same settings as **c**

The *K* and ${\Delta }_{C}$ estimates which deviate from their true values by AUS effects are denoted as AUS *K* and AUS ${\Delta }_{C}$ in Fig. 4. Figure 4a, b show the individual effects of *a*_1_ and *a*_2_, respectively, on AUS *K* and AUS ${\Delta }_{C}$. In Fig. 4a, $a_{1}$ itself causes no deviation for AUS $K$ but it raises the values of AUS ${\Delta }_{C}$. In Fig. 4b, increase of $a_{2}$ causes a greater reduction in AUS $K$ but again raises the AUS ${\Delta }_{C}$ values. The observed trends suggest that the experimentally estimated $K$ is likely smaller than the true $K$ whereas the experimentally estimated ${\Delta }_{C}$ is likely greater than the true ${\Delta }_{C}$.

Figure 4c, d show the simulation of collective AUS effects and ring current effects on *K* and ${\Delta }_{C}$ estimates for offset complex geometries, Fig. 2. In Fig. 4c, reduction in AUS K is enhanced by weaker ring current effects (smaller set ${\Delta }_{C}$) and by stronger AUS effects. For example, AUS *K* at ${\Delta }_{C} = 0.8$ reduces from 0.776 to 0.607 when *a*_2_ increases from $0.01$ to $0.09$ (the first curve near the top of Fig. 4c). When ${\Delta }_{C}$ is set as 0.01, AUS *K* exhibits remarkable reduction from 0.635 to 0.029 within the tested $a_{2}$ value range. As for ${\Delta }_{C}$ estimates weaker ring current effects (smaller set ${\Delta }_{C}$ values) enhances AUS effects on deviation of AUS ${\Delta }_{C}$ from true values. In Fig. 4d we can see that the increment of AUS ${\Delta }_{C}$ per *a*_2_ unit increases with smaller set ${\Delta }_{C}$ values, especially when ${\Delta }_{C}$ value is set smaller than 0.1. For example, at $a_{2} = 0.09,$ AUS ${\Delta }_{C}$ is 1.176 which is 0.376 more than the set value 0.8. When the set ${\Delta }_{C}$ value becomes 0.01, AUS ${\Delta }_{C}$ exhibits a drastically increased value 3.319. The simulation suggests not only that the ${\Delta }_{C}$ is overestimated under the influences of both AUS effects and small ring current effects but the protons that far away from stacking donors could give extremely large estimate of ${\Delta }_{C}$.

**Fig. 5:**
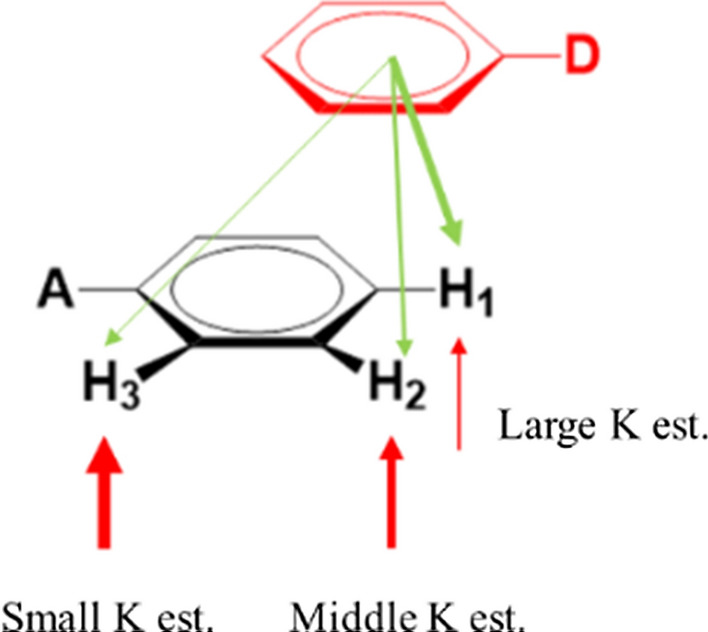
The relationship among *K* estimates (*K *est. in the figure), AUS effect (*a*_2_ effect presented in red arrows) and ring current effects (green arrows). Larger weight of arrows means stronger AUS effects

Our simulation results unravel the deviation patterns of *K* and ${\Delta }_{C}$ estimation from the true values in the presence of AUS effects when offset *π*–*π* complexes are studied. In general, AUS effects tend to induce underestimation of true *K* and overestimation of true ${\Delta }_{C}$. For an offset *π*–*π* complex the protons of an acceptor can sense different degrees of ring current effects and AUS effects, Fig. 5. The acceptor protons away from the stacking donor likely sense weaker ring current effects (smaller true ${\Delta }_{C}$ values) and stronger AUS effects (greater *a*_2_) due to more free donor collisions [24]. According to our analysis, these protons tend to exhibit smaller *K* estimates due to greater underestimation, Fig. 5. In other words, the protons closer to the stacking donor are likely to exhibit larger $K$ estimates due to the least underestimation. Therefore, various degrees of AUS effects and ring current effects {} at the protons of an offset complex not only lead to underestimation of true $K$, but also result in the different $K$ estimates. The values of the different $K$ estimates depend on the positions of observed protons relative to the stacking donor. For experimentally obtained $K$ estimates we, for the first time, propose that the largest estimate may be selected to be a better $K$ estimate than the average one for *π*–*π* complexes due to its least deviation from the true $K$ and the variation in $K$ estimates could be used to infer the complex geometries due to highly geometric dependence.

**Fig. 6:**
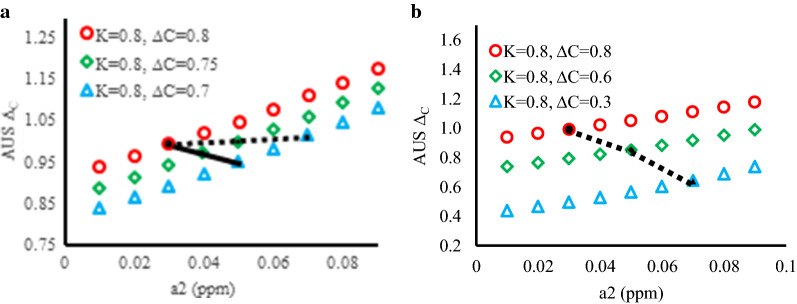
The size orders of AUS $${{\varvec{\Delta}}}_{{\varvec{C}}}$$ do not always match those of the set $${{\varvec{\Delta}}}_{{\varvec{C}}}$$. Assume that an offset complex has three set $${{\varvec{\Delta}}}_{{\varvec{C}}}$$ values. The larger set $${{\varvec{\Delta}}}_{{\varvec{C}}}$$ values correspond to smaller $${\varvec{a}}_{2}$$ due to less AUS effects. The resulting three AUS $${{\varvec{\Delta}}}_{{\varvec{C}}}$$ values are connected with lines. a the data points in the dashed line: ($${\varvec{a}}_{2} = 0.03$$, AUS $${{\varvec{\Delta}}}_{{\varvec{C}}} = 0.992$$), ($${\varvec{a}}_{2} = 0.05$$, AUS $${{\varvec{\Delta}}}_{{\varvec{C}}} = 1.000$$) and ($${\varvec{a}}_{2} = 0.07$$, AUS $${{\varvec{\Delta}}}_{{\varvec{C}}} = 1.014$$). The data points in the solid line are ($${\varvec{a}}_{2} = 0.03$$, AUS $${{\varvec{\Delta}}}_{{\varvec{C}}} = 0.992$$), ($${\varvec{a}}_{2} = 0.04$$$$,$$ AUS $${{\varvec{\Delta}}}_{{\varvec{C}}} = 0.970$$) and ($${\varvec{a}}_{2} = 0.05$$, AUS $${{\varvec{\Delta}}}_{{\varvec{C}}} = 0.951$$); b the data points in the dashed line: ($${\varvec{a}}_{2} = 0.03$$$$,$$ AUS $${{\varvec{\Delta}}}_{{\varvec{C}}} = 0.992$$), ($${\varvec{a}}_{2} = 0.05$$, AUS $${{\varvec{\Delta}}}_{{\varvec{C}}} = 0.852$$ ) and ($${\varvec{a}}_{2} = 0.07$$, AUS $${{\varvec{\Delta}}}_{{\varvec{C}}} = 0.644$$)

In terms of ${{\varvec{\Delta}}}_{{\varvec{C}}}$, our simulation results show that the true ${{\varvec{\Delta}}}_{{\varvec{C}}}$ values tend to be overestimated in the presence of AUS effects. Weaker ring current effects could also enhance this overestimation. However, unlike ***K*** estimates, ${{\varvec{\Delta}}}_{{\varvec{C}}}$ estimates may not show a clear relationship with their true values due to complicated interactions among the collective effects on ${{\varvec{\Delta}}}_{{\varvec{C}}}$ overestimation. For example, three set ${{\varvec{\Delta}}}_{{\varvec{C}}}$ values (0.8, 0.75 and 0.7 in Fig. 4d presented in Fig. 6a) at ${\varvec{a}}_{2}$ values (0.03, 0.05, 0.07) correspond to the AUS ${{\varvec{\Delta}}}_{{\varvec{C}}}$ values, 0.992, 1.000, 1.014, respectively, which exhibits a inversely proportional size relationship between set ${{\varvec{\Delta}}}_{{\varvec{C}}}$ values and AUS ${{\varvec{\Delta}}}_{{\varvec{C}}}$ values. However, in the same Fig. 6, the AUS ${{\varvec{\Delta}}}_{{\varvec{C}}}$ values at more alike ${\varvec{a}}_{2}$ values (0.03, 0.04, 0.05) for the set ${{\varvec{\Delta}}}_{{\varvec{C}}}$ values (0.8. 0.75, 0.7) are 0.992, 0.970 and 0.951, respectively, which is a directly proportional size relationship. Likewise, in Fig. 6b, the ${{\varvec{\Delta}}}_{{\varvec{C}}}$ estimates at ${\varvec{a}}_{2}$ values (0.03, 0.05, 0.07) for more distinct set ${{\varvec{\Delta}}}_{{\varvec{C}}}$ values (0.8, 0.6, 0.3) are 0.992, 0.852 and 0.644, respectively. Considering the difficulty of estimation of AUS effects and unknown true ${{\varvec{\Delta}}}_{{\varvec{C}}}$ values at each observed proton, it is not easy to judge which relationships between estimated ${{\varvec{\Delta}}}_{{\varvec{C}}}$ and true ${{\varvec{\Delta}}}_{{\varvec{C}}}$ is encountered, so that it may raise concerns when ${{\varvec{\Delta}}}_{{\varvec{C}}}$ estimates are used to infer complex geometries.

## Discussion

### Inferences for association constants

Our simulation provides clear clues to understand the influence of AUS effects on *K* and ${\Delta }_{C}$ estimates under general experimental conditions when the protons in offset complex geometries are investigated. According to the simulation results, it is easy to interpret the obtained *K* listed in Table 1. The true *K* of each complex is underestimated to different degrees depending on the observed protons. The largest values among the obtained *K* estimates of any complex in Table 1 could be selected as the best complex *K* estimate. Even for the TA complex where we do not find strong evidence to distinguish its two *K* estimates, the selection of the larger *K* estimate might still provide equal or better estimation than the averaged one.

As for the Py complex, the curve fitting yields three small *K* values. However, our bootstrap analysis shows that the negative percentile range, − 0.058 and − 0.017 M^−1^, for *K* and the range, − 2.83 and − 0.81 ppm, for ${\Delta }_{C}$ at ortho protons (H2 and H6) despite that such negative values are not seen in the regular data treatment, Table 1. For the near zero or negative *K* values Stamm et al. suggest that, for complexes with near zero *K*, the upfield shift data of protons experiencing negligible ring current effects usually present no relationship (horizontal lines) or a linear relationship with positive slope in the plot of ${\Delta }/d_{0}$ vs ${\Delta }$ [28] leading to near zero or negative *K* estimates. We utilized this plot as a diagnosis tool to analyze all upfield shift data in this study and found that all data exhibit linear relationships with negative slopes that are significantly different from the horizontal line (significant level $\alpha = 0.05$) except for the data at ortho protons (H2, H6) of the Py complex which display slightly positive slopes. This may correspond to the negative value range of *K* in our bootstrap results. Therefore, the *K* estimate of Py ortho protons in Table 1 was neglected and the *K* estimate 0.082 M^−1^ for the Py para protons (H4) is selected for the Py complex.

### Inferences for complex geometries

**Fig. 7:**
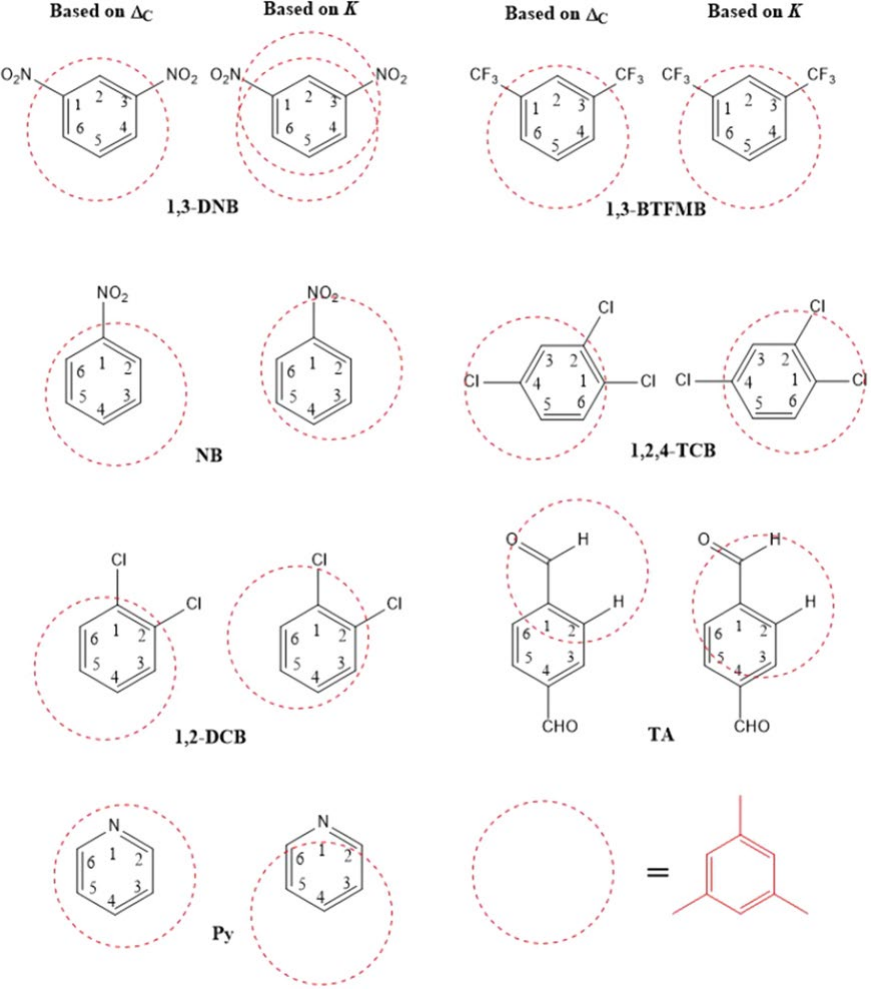
The geometric inferences for tested acceptors based on two approaches: the first uses the ${{\varvec{\Delta}}}_{{\varvec{C}}}$ order based on the values estimated by global curve fitting in Table 1; the second uses the ***K*** orders directly from individual curve fitting, Table 1. Avoiding the confusion, MSTL is represented by the red dotted circles used to approximate the likely locations of MSTL on acceptors

The geometries of *π*–*π* complexes in solvents have been one of hot research topics, especially for relatively weak complexes. Estimates of ${\Delta }_{C}$ are commonly used in history to infer relative positions of protons of an acceptor to the complexed donor [19, 28]. Nevertheless, with the robust bootstrap technique and the numerical simulation for AUS effects, we found that larger ${\Delta }_{C}$ estimates could correspond to either larger or smaller true ${\Delta }_{C}$ values. Therefore, the ${\Delta }_{C}$-based geometric inferences may be unreliable. On the contrary, the *K* estimates are highly associated with geometrically positioned protons. The protons with larger *K* estimates are expected to be closer to the stacking donor, Fig. 5. Here we present the results of geometric inferences in Fig. 7 using two methods: based on ${\Delta }_{C}$ estimates and based on *K* estimates in Table 1. In general, the method based on *K* estimates provides preferable geometric inferences as compared to those based on ${\Delta }_{C}$ estimates when substituent effects and steric hindrance reported in various literatures are considered (see the inference description below). It is noticeable that two associated rings might slide along ring surfaces to give various configurations for offset geometries [39]. The inferences only reflect the most likely geometries for complexes.

In Fig. 7, 1,3-DNB has the ${\Delta }_{C}$ estimates in the order: H5 > (H4, H6) > H2 which indicates that MSTL associates closer to H5 of 1,3-DNB. On the other hand, 1,3-DNB has the *K* estimates in the order: H2 > H5 > (H4, H6) which shows two possible complex isomers, one near H2 and the other near H5. Stacking of MSTL near H2 of 1,3-DNB may look counterintuitive because this region is flanked by two nitro groups, which exhibits certain steric hindrance due to protrusion of the partial negatively charged oxygen moieties on the nitro groups through torsional vibration [19]. However, the nitro groups also cause an electron poor region near H2 of 1,3-DNB and provide London dispersion force through their $\pi$ bonds, both of which can stabilize the $\pi$ cloud of stacking rings [40]. With the similar structure but much weaker and bulky substituents at the position 1 and 3, 1,3-BTFMB complexes only shows one possible geometry. Both *K* and the ${\Delta }_{C}$ estimates display the same order: H5 > (H4, H6) > H2 which suggests that MSTL associates near H5 of 1,3-BTFMB.

For NB, 1,2,4-TCB and 1,2-DCB complexes, MSTL tends to stack in close proximity to substituents based on their *K* estimates whereas it tends to stack away from the substituents based on their ${\Delta }_{C}$ orders. For NB complexes, MSTL stacks closer to the meta protons by the ${\Delta }_{C}$ order but near ortho protons by the *K* order. For 1,2,4-TCB, MSTL stacks near H5 by the ${\Delta }_{C}$ order but near H6 by the *K* order. For 1,2-TCB complex, MSTL may stack near H4 or H5 by the ${\Delta }_{C}$ order. Using the *K* order, we consider that MSTL may stack near either H3 or H6. Considering the steric hindrance of substituents, it might be more intuitively acceptable that MSTL associates away from chloro or nitro groups of acceptors. Nevertheless, Sherrill et al.’s [40] theoretical study generally shows more energy favorable association of benzene in proximity to the ring region near acceptor substituents than the ring region opposite to the substituens, which supports the geometries inferred by the *K* orders.

For TA complexes, the ${\Delta }_{C}$ order indicates a stacking position of MSTL near aldehyde protons and away from the ring of TA. However, the geometry based on the *K* order suggests a stacking position near both the aldehyde protons and the ortho protons of the TA ring. The latter geometry is better supported by Sherrill et al.’s theoretical studies due to more participation of a ring in *π*–*π* assembly [40, 41]. In fact, this geometry also resembles the assembly of toluene and the rings with aldehyde groups reported by Stamm et al. [28].

For Py complexes, the geometry based on the ${\Delta }_{C}$ order indicates that MSTL associates near H3 or H5 with large ring contact due to three large ${\Delta }_{C}$ estimates but that based on *K* estimates suggests the association in close proximity to H4 and far away from ortho protons with less ring contact. Py is usually used as a donor in the field of charge transfer complexes [42, 43]. The geometry is expected to be loose and offset due to the repulsion between $\pi$ clouds of Py and MSTL resulting in both small true *K* and ${\Delta }_{C}$. In our experiments, the Py complex exhibits the smallest *K* estimates but largest ${\Delta }_{C}$ estimates among the tested complexes. The small *K* estimates reflect on the weak stability of Py complexes. The large ${\Delta }_{C}$ estimates could be explained by our numeric simulation. The simulation shows that very small true ${\Delta }_{C}$ tends to cause greater degree of ${\Delta }_{C}$ overestimation through AUS effects. Moreover, the theoretical study shows that the region of Py ring near H3, H4 and H5 is relatively more electron deficient than that near its nitrogen [44]. The region near H4 provides slight association attraction for stacking of MSTL molecule, which is better predicted by the *K* order.

## Conclusion

Proton-NMR is the simplest and the most useful tool for analysis of weak *π*–*π* complexes in liquid. However, the proton-based analysis is limited by often-encountered confusing results including various *K* estimates and unreasonable large ${\Delta }_{C}$ estimates for weak *π*–*π* complexes. Historically treatment for such an issue is to simply ascribe the difference to experimental errors and to use average *K* for evaluation of stacking stability and ${\Delta }_{C}$ estimates for inference of complex geometries. However, with a robust bootstrap statistical method, we found that the difference in *K* is better interpreted by the different contribution of excess free donors to the upfield shifts of the protons of an offset complex due to AUS effects. Our numerical simulation shows that true *K* tends to be underestimated and true ${\Delta }_{C}$ tends to be overestimated. The best estimate of true *K* for a *π*–*π* complex could be obtained by selecting the largest $K$ estimate and the geometries could be reasonably inferred by comparing highly geometric-dependent *K* estimates rather than ${\Delta }_{C}$ estimates. The outcome of this report is expected to improve proton-NMR analysis for interpretation of weak *π*–*π* complexation in liquid and contribute to the applications of relevant research fields such as supramolecular chemistry, conformation of biomolecules and quantitative analysis of aromatic related drugs.

## Supplementary information

**Additional file 1.** The explanation for Eq. 2 development.

## Data Availability

All data is included within this article.

## Abbreviations

1,3-DNB: 1,3-Dinitrobenzene
NB: Nitrobenzene
1,3-BTFMB: 1,3-Bis(trifluoromethyl) benzene
1,2,4-TCB: 1,2,4-Trichlorobenzene
1,2-DCB: 1,2-Dichlorobenzene
TA: Terephthalaldehyde
Py: Pyridine
MSTL: Mesitylene
Proton-NMR: Proton-nuclear magnetic resonance
AUS effects: Additional unspecific shielding effects
*K*: Association constants
$${\Delta}_{C}$$: Complex shifts
SE: Standard errors
