# Supplementary material for: New insights into prediction of weak π–π complex association through proton-nuclear magnetic resonance analysis
Source: BMC Chem. 2020 Oct 30;14(1):66. doi: 10.1186/s13065-020-00718-x (PMC7602360; doi:10.1186/s13065-020-00718-x)
Supplement: Supplementary file 1 — Additional file 1. The explanation for Eq. 2 development. [file 13065_2020_718_MOESM1_ESM.docx]

Additional Material for “New Insights into Prediction of Weak π-π Complex Association through Proton-Nuclear Magnetic Resonance Analysis”

Chenyu Lin,^a^*^*^* Joseph Skufca^b^ and Richard E. Partch*^c^*

^a,c^ Chemistry & Biomolecular Department, Clarkson University, Potsdam, New York, 13699, USA

^a^ chenyuchemist@gmail.com

^c^ rpartch@clarkson.edu

^b^ Mathematics Department, Clarkson University, 8 Clarkson Ave., Potsdam, New York, 13699, USA

^b^ [jskufca@clarkson.edu](mailto:jskufca@clarkson.edu)

**The explanation for Eq. 2 development**

For a pi-pi complexation of an acceptor and a donor, the association constant K can be expressed as the following

$K=\frac{c}{(a_{0}-c){(d}_{0}-c)}$ Eq. S1

Where $c, a_{0}$ and $d_{0}$ are the complex concentrations, initial acceptor concentrations, and initial donor concentration, respectively.

The association constant equation can be solved for c as

$c=\frac{\left( Ka_{0}+Kd_{0}+1 \right)-\sqrt{\left( Ka_{0}+Kd_{0}+1 \right)^{2}-4K^{2}a_{0}d_{0}}}{2K}$ Eq. S2

Without the influence of AUS effect, the relationship among the observed chemical shift ($\delta$) in the presence of donors, the chemical shift of non-complexed acceptor protons ($\delta_{A}$) and the acceptor protons in a complex ($\delta_{C}$) can be expressed as:

$\delta_{A}-\delta=\frac{c}{a_{0}}(\delta_{A}-\delta_{C})$ Eq. S3

Under the AUS influence (Figure 3), the $\delta_{A}$ and $\delta_{C}$ would be shift to the right by $a_{1}d_{0}$ and $a_{2}d_{0}$, respectively, which lead to

$\delta_{A}-a_{1}d_{0}-\delta=\frac{c}{a_{0}}((\delta_{A}-a_{1}d_{0})-(\delta_{C}-a_{2}d_{0})$) Eq. S4

Then this equation can be rearranged as

$\delta_{A}-\delta=\frac{c}{a_{0}}\left( \left( \delta_{A}-\delta_{C} \right)-a_{1}d_{0}+a_{2}d_{0} \right)+a_{1}d_{0}$ Eq. S5

Considering $\delta_{A}-\delta=\Delta$, $\delta_{A}-\delta_{C}=\Delta_{C}$, and Eq. S2, the Eq. S5 can be transformed into Eq. 2 used in the manuscript:

$$\Delta=\frac{\left( Ka_{0}+Kd_{0}+1 \right)-\sqrt{\left( Ka_{0}+Kd_{0}+1 \right)^{2}-4K^{2}a_{0}d_{0}}}{2a_{0}K}\left( \Delta_{C}-a_{1}d_{0}+a_{2}d_{0} \right)+a_{1}d_{0}$$
